# Using Video Calls to Reduce Risky Behaviors and Enhance Instruction Understanding of Patients in Acute Care Hospitals During the COVID-19 Pandemic

**DOI:** 10.7759/cureus.45074

**Published:** 2023-09-12

**Authors:** Kazuya Watanabe, Takuaki Tani, Atsushi Suzuki, Kei Kawakami, Mami Watanabe, Kei Yamasaki, Koichi Morota

**Affiliations:** 1 Department of Physical Therapy, Shimonoseki Nursing and Rehabilitation School, Yamaguchi, JPN; 2 Department of Psychosomatic Dentistry, Tokyo Medical and Dental University Graduate School of Medical and Dental Sciences, Tokyo, JPN; 3 Division of Pharmacoepidemiology, Department of Healthcare and Regulatory Sciences, Showa University School of Pharmacy, Tokyo, JPN; 4 Department of Rehabilitation, Shinkomonji Hospital, Fukuoka, JPN; 5 Department of Radiology, Shinkomonji Hospital, Fukuoka, JPN

**Keywords:** acute care hospitals, covid-19, enhance instruction understanding, risky behaviors, video calls

## Abstract

Backgrounds

During the COVID-19 pandemic, visitor restrictions in healthcare settings adversely affected patients. Video calls have emerged as an essential digital alternative that can decrease patients’ anxiety and improve satisfaction. This study investigated whether family-initiated video calls could mitigate delirium symptoms and risky behaviors and enhance patients’ comprehension of instructions.

Methods

This observational study used medical chart data and the Diem Payment System from a single acute care hospital in Fukuoka, Japan. The study involved patients hospitalized between May 2020 and August 2021 who used video chat systems. Patients or their relatives used video calls through Skype. The frequency of video chat use served as the primary exposure. Changes in the patients' risky behaviors and instruction comprehension upon discharge were the primary outcomes.

Results

A total of 532 patients were included in the study, with an average age of over 70 years. After implementing the inverse probability of treatment weighting adjustment, an improved balance across age, sex, BMI categories, and other variables was observed. The effects of video calls on risky behaviors and instruction comprehension varied. Patients with three or more video calls showed distinct effects compared with those with fewer calls. When hospitalization was limited to three weeks, video calls noticeably influenced risky behaviors (p=0.022, 95% CI:1.08-2.63), but not instruction comprehension (p=0.226, 95% CI:0.43-1.22).

Conclusions

The use of video calls as a visitation method in acute care hospitals during a pandemic suggests that video calls reduce risky behaviors in patients with a three-week stay. This alternative to physical visitations contributes positively to patient safety and supports ongoing efforts to prevent the spread of COVID-19.

## Introduction

In response to the coronavirus disease 2019 (COVID-19) outbreak, medical facilities implemented visitation restrictions to mitigate the spread of the virus [1]. These limitations have led to a range of physical health implications for patients, including decreased nutritional intake, reduced activities of daily living, and heightened physical distress. Significant effects on mental health, including increased loneliness, depressive symptoms, anger, anxiety, and overall dissatisfaction, have also been reported [1,2]. Video call visits have become increasingly important for counteracting restrictions on in-person visits [3]. This digital approach, which numerous healthcare providers now use, facilitates the interaction between patients and their families [3].

Existing research has shown a strong correlation between pandemic-related visitation restrictions and a high incidence of delirium among patients with acute cerebrovascular disease in stroke units [4]. This connection is observed among emergency inpatients regardless of age, ward assigned to upon admission, ventilator management, general anesthesia surgery, and dementia [5]. In surgical patients, visitation restrictions lead to increased disorientation, insomnia, and the self-removal of medical devices [6].

Among these are increased patient risk behaviors such as self-extraction of intravenous used in treatment, falls, and self-injurious behavior, as well as poor understanding of instructions, which hinder recovery from illness and injury. These are serious problems that increase the nursing burden on nurses who have the most contact with patients during hospitalization.

Video call visits provide tangible benefits to patients and their families, including increased satisfaction, increased motivation, reduced anxiety, decreased depressive symptoms, and reduced distress [7,8]. Especially for older people, video calls provide an important link to family members and effectively reduce loneliness and social isolation which are prevalent in care settings [9]. Video call visits have been reported as an alternative to face-to-face visits [7]. However, the effect of the frequency of video calls on patients' outcomes remains unknown.

This study aimed to ascertain whether the frequency of video calls, acting as a substitute for in-person visits due to visitation restrictions in acute care hospitals, is associated with an improvement in patients' risk behaviors related to delirium symptoms and instruction comprehension during the COVID-19 pandemic.

## Materials and methods

Study design

This observational study was a retrospective study using Diagnosis Procedure Combination (DPC) and medical record data of patients at a single hospital in Japan.

Data source

Diagnosis Procedure Combination Per-Diem Payment System data and medical chart data from a high acute care hospital in Fukuoka, Japan, were used. The Diagnosis Procedure Combination Per-Diem Payment System is a national acute-care inpatient database in Japan that provides administrative claims data for a fixed payment system for medical expenses [10]. The database includes patient information, such as age, sex, weight, height, and length of hospitalization, and disease information, such as main diagnoses, comorbidities, and complications, coded using the International Classification of Diseases 10th Revision (ICD-10) [11]. The database also includes admission information such as whether patients were admitted to an intensive care unit (ICU) or high care unit (HCU); information on medical procedures such as the use of anesthesia; patient status information, including information on whether risk behavior is present and whether patients understand instructions; and patients’ results on the Japan Coma Scale, which is commonly used to measure coma status at admission in Japan [12]. The medical chart data contained information on when and how many video calls were conducted and to which ward the patient was admitted.

Patient selection

This study analyzed the data of patients hospitalized at Shinkomonji Hospital between May 2020 and August 2021 who used a video chat system.

Outcomes

The primary outcomes were risky behavior and understanding of instructions, each evaluated as a binary value of presence or absence. Risky behavior was defined as self-removal of intravenous infusions during treatment, falls, self-injurious behavior, and any other behavior that nurses or others judged to be potentially risky. If there has been no hazardous behavior within the past week or there is no record of such behavior, the patient is rated as "none". If the patient has engaged in risky behavior within the past week and there is a record of such behavior, it is rated as "Yes". The understanding of instructions is evaluated based on whether the patient is able to understand and carry out medical and treatment instructions, regardless of the disease in question. The evaluation criterion is "none" if the patient always acts appropriately in response to medical and treatment instructions, or if there is no record of actions not taken in accordance with instructions. The evaluation criteria are as follows: "Yes" if the patient has at least one instance of behavior that is not in accordance with medical or medical treatment instructions, and if there is a record of the behavior that is not in accordance with the instructions [13]. These outcomes were assessed daily by the charge nurse and recorded in the electronic medical record.

Video chat

Video calls for visitations were made upon request from patients or their families. The patient was accompanied by a nurse in his room, and the family used the visiting room in the ward. Each visitation session lasted for 20 min. Skype was used for video calls; Skype uses Voice over Internet Protocol technology to communicate using voice and video over a real-time connection via the Internet [14]. Visits were allowed only in the afternoon by appointment, with a limit of one visit per day and a limit of seven visits per week.

Other variables

We extracted information on age, sex, length of stay, destination of discharge, use of general anesthesia, and ward admitted to (oncology, urology, respiratory, orthopedic, gastrointestinal, or cardiovascular). We calculated body mass index (BMI) as weight (kg) divided by height squared (m^2^) and created five categories. BMI category was assigned based on the modified World Health Organization classifications of <18.5 kg/m^2^, 18.5-24.9 kg/m^2^, 25.0-29.9 kg/m^2^, 30.0-34.9 kg/m^2^, ≥35 kg/m^2^ [15-16]. The Charlson comorbidity index (CCI) was calculated based on Quan's protocol using the ICD-10 [11,17]. Dementia was coded according to ICD-10 [11]. We extracted medication information from HCUs and ICUs.

Statistical analyses

Inverse probability of treatment weighting (IPTW) was used to adjust for differences in baseline characteristics and to estimate the average treatment effect using probability weights assigned to video chat frequencies of one, two, three, and ≥four times [18].

The weights used in the IPTW were determined using the propensity scores (PSs). PSs were created using a multivariate logistic regression model to predict the probability of video chat frequencies of one, two, three, and ≥four times. IPTW values were calculated using the baseline characteristics of each patient to estimate the average treatment effect [19]. The variables, adjusted for confounders, included age, BMI, CCI score, Japan Coma Scale (JCS) score, use of anesthesia, ICU and HCU admission, and ward admitted to.

After weighing the baseline, baseline covariates were compared using absolute mean differences (AMDs) [19]. To compare the performances of the two groups before and after stabilized IPTW, AMD > 10% was regarded as an imbalance. We used a generalized linear model with a log-link function and quasi-Poisson distribution to estimate the efficacy of video chat frequency on patient status, whether risk behavior was present, and whether instructions were understood at discharge.

Missing values were handled by multiple imputations using chained equations [20] generating 20 multiple imputation datasets and conducting the main analysis for each dataset. The results were combined using Rubin's rule to obtain the final estimates and 95% confidence intervals (CIs). A two-sided p-value of <0.05 and a standardized mean difference (SMD) of <0.1 was considered statistically significant. All analyses were performed using R software version 4.2.2.

Sensitivity analysis

We conducted sensitivity analyses. The length of hospital stay was limited to the number of days within three weeks (median length of hospital stay for participants). Maybe, the longer the length of hospital stay, the more effective the inference from the video chats. Moreover, the longer the length of hospital stay, the greater the effect observed in the patient’s status on whether risky behavior is present and whether instructions are understood at discharge.

This study was approved by the Clinical Research Ethics Committee of Shinkomonji Hospital (Approval ID:2021009). The retrospective and anonymized nature of the data exempted the study from requiring explicit participant consent [21].

## Results

A total of 756 patients who were admitted to Shinkomonji Hospital from May 2020 to August 2021 used Skype. Of these, 532 patients with information on the number of times they used Skype were included in the study.

Tables 1-3 show the patient demographics before and after the application of the IPTW adjustment. The standardized mean difference (SMD) was used to assess the balance of baseline covariates across the treatment groups. Before adjustment, the mean age of patients who engaged in video calls differed slightly among groups, ranging from 79.23 to 81.64 years. However, after the IPTW adjustment, the mean age exhibited a smaller variation, extending from 80.19 to 81.15 years, with a decrease in the SMD from 0.106 to 0.038. This indicates an improved balance in the age distribution across the groups. Regarding sex, the percentage of women in each group varied from 57.3% to 65.7% before adjustment, with an SMD of 0.093. After adjustment, this range narrowed to between 57.9% and 59.6%, and the SMD decreased to 0.018, suggesting a more balanced sex distribution. Regarding BMI distribution, both the adjusted and unadjusted analyses showed similar trends, with ‘normality’ being the most prevalent category, succeeded by ‘underweight’, ‘overweight’, and ‘obesity’. The SMD decreased from 0.177 to 0.092 after adjustment, indicating an improved balance in BMI categories across the groups. The CCI total score at admission, JCS score at admission, history of general anesthesia, dementia, history of ICU and HCU admissions, length of stay, and type of inpatient unit all showed reduced SMDs post-adjustment, implying an improved balance of these variables across the treatment groups following IPTW application. In summary, the IPTW adjustment led to a better balance across the covariates, making the treatment groups more comparable and strengthening the validity of further causal inferences drawn from the data.

**Table 1:** Patient demographics before and after adjustment using IPTW IPTW: Inverse Probability of Treatment Weighting, SMD: Standardized Mean Difference, SD: Standard Deviation, BMI: Body Mass Index

	Without adjustment						IPTW with adjustment				
	1 time	2 times	3 times	more than 4 times	SMD		1 time	2 times	3 times	more than 4 times	SMD
	(n=249)	(n=106)	(n=67)	(n=110)			(n=529.78)	(n=545.04)	(n=528.85)	(n=530.26)	
Age(y), (mean ± SD)	81.64 ± 11.90	79.70 ± 14.35	80.73 ± 13.20	79.23 ± 13.25	0.106		80.90 ± 12.84	80.19 ± 13.47	81.15 ± 12.40	80.99 ± 12.53	0.038
Sex (female), n (%)	148 (59.4)	61 (57.5)	44 (65.7)	63 (57.3)	0.093		315.9 (59.6)	321.7 (59.0)	311.9 (59.0)	307.0 (57.9)	0.018
BMI, n (%)					0.177						0.092
normality	140 (56.2)	63 (59.4)	37 (55.2)	66 (60.0)			314.3 (59.3)	327.0 (60.0)	290.5 (54.9)	314.8 (59.4)	
overweight	31 (12.4)	20 (18.9)	9 (13.4)	16 (14.5)			69.9 (13.2)	63.5 (11.7)	87.1 (16.5)	71.7 (13.5)	
obesity	14 (5.6)	2 (1.9)	3 (4.5)	5 (4.5)			22.4 (4.2)	25.9 (4.8)	19.4 (3.7)	18.1 (3.4)	
underweight	64 (25.7)	21 (19.8)	18 (26.9)	23 (20.9)			123.1 (23.2)	128.7 (23.6)	131.8 (24.9)	125.7 (23.7)	
Dementia, n (%)	133 (53.4)	56 (52.8)	38 (56.7)	65 (59.1)	0.074		293.1 (55.3)	285.7 (52.4)	264.9 (50.1)	289.9 (54.7)	0.06

**Table 2:** The Charlson Comorbidity Index and Japan Coma Scale score before and after adjustment using IPTW IPTW: Inverse Probability of Treatment Weighting, SMD: Standardized Mean Difference, SD: Standard Deviation, CCI: Charlson Comorbidity Index, JCS: Japan Coma Scale

	Without adjustment						IPTW with adjustment				
	1 time	2 times	3 times	more than 4 times	SMD		1 time	2 times	3 times	more than 4 times	SMD
	(n=249)	(n=106)	(n=67)	(n=110)			(n=529.78)	(n=545.04)	(n=528.85)	(n=530.26)	
CCI total score at admission, n (%)					0.248						0.072
0	79 (31.7)	36 (34.0)	21 (31.3)	37 (33.6)			175.5 (33.1)	180.2 (33.1)	166.1 (31.4)	186.3 (35.1)	
1	67 (26.9)	31 (29.2)	19 (28.4)	30 (27.3)			142.7 (26.9)	147.0 (27.0)	144.9 (27.4)	143.6 (27.1)	
2	59 (23.7)	25 (23.6)	8 (11.9)	23 (20.9)			115.1 (21.7)	114.3 (21.0)	119.8 (22.6)	105.9 (20.0)	
3	25 (10.0)	7 (6.6)	11 (16.4)	11 (10.0)			54.3 (10.3)	48.5 (8.9)	48.4 (9.1)	49.5 (9.3)	
4＞	19 (7.6)	7 (6.6)	8 (11.9)	9 (8.2)			42.1 (8.0)	55.0 (10.1)	49.7 (9.4)	45.0 (8.5)	
JCS at admission, n (%)					0.334						0.086
0	145 (58.2)	54 (50.9)	29 (43.3)	50 (45.5)			280.3 (52.9)	287.0 (52.7)	287.3 (54.3)	272.6 (51.4)	
1	84 (33.7)	45 (42.5)	31 (46.3)	38 (34.5)			188.6 (35.6)	186.3 (34.2)	188.2 (35.6)	192.9 (36.4)	
2	13 (5.2)	5 (4.7)	4 (6.0)	6 (5.5)			32.4 (6.1)	33.3 (6.1)	34.0 (6.4)	36.0 (6.8)	
3	7 (2.8)	2 (1.9)	3 (4.5)	16 (14.5)			28.5 (5.4)	38.4 (7.1)	19.4 (3.7)	28.8 (5.4)	

**Table 3:** Patient admission information before and after adjustment using IPTW IPTW: Inverse Probability of Treatment Weighting, SMD: Standardized Mean Difference, SD: Standard Deviation, ICU: Intensive Care Unit, HCU: High Care Unit

	Without adjustment						IPTW with adjustment				
	1 time	2 times	3 times	more than 4 times	SMD		1 time	2 times	3 times	more than 4 times	SMD
	(n=249)	(n=106)	(n=67)	(n=110)			(n=529.78)	(n=545.04)	(n=528.85)	(n=530.26)	
Type of Inpatient Unit, n (%)					0.414						0.141
Internal medicine	19 (7.6)	7 (6.6)	3 (4.5)	5 (4.5)			35.0 (6.6)	35.3 (6.5)	36.9 (7.0)	30.0 (5.7)	
Urological	10 (4.0)	10 (9.4)	3 (4.5)	4 (3.6)			26.5 (5.0)	28.0 (5.1)	29.3 (5.5)	27.3 (5.1)	
Respiratory surgery	39 (15.7)	11 (10.4)	8 (11.9)	7 (6.4)			64.2 (12.1)	59.9 (11.0)	87.5 (16.5)	61.5 (11.6)	
Other	31 (12.4)	12 (11.3)	6 (9.0)	21 (19.1)			75.5 (14.3)	85.2 (15.6)	52.7 (10.0)	71.5 (13.5)	
Orthopedics	78 (31.3)	25 (23.6)	26 (38.8)	29 (26.4)			156.7 (29.6)	156.3 (28.7)	169.1 (32.0)	167.5 (31.6)	
Digestive surgery	23 (9.2)	12 (11.3)	3 (4.5)	6 (5.5)			42.1 (7.9)	39.5 (7.2)	39.9 (7.5)	43.2 (8.1)	
Life-saving	49 (19.7)	29 (27.4)	18 (26.9)	38 (34.5)			129.7 (24.5)	140.8 (25.8)	113.5 (21.5)	129.3 (24.4)	
History of general anesthesia, n (%)	75 (30.1)	23 (21.7)	18 (26.9)	46 (41.8)	0.232		165.1 (31.2)	175.8 (32.3)	145.9 (27.6)	159.6 (30.1)	0.055
History of ICU or HCU admissions, n (%)	31 (12.4)	22 (20.8)	15 (22.4)	25 (22.7)	0.143		89.8 (17.0)	90.1 (16.5)	94.3 (17.8)	93.9 (17.7)	0.02
Length of stay, (mean ± SD)	22.94 ± 22.55	24.88 ± 25.62	28.31 ± 17.85	36.44 ± 22.33	0.331		23.17 ± 22.79	26.03 ± 25.44	29.65 ± 18.92	35.22 ± 22.45	0.296

Table 4 presents a breakdown of the frequency of video calls relative to the improvement in instructional understanding and the occurrence of dangerous behavior. Of the 481 patients who showed no improvement, 223 (46.4%) used video calls once, 98 (20.4%) used them twice, 62 (12.9%) used them thrice, and 98 (20.4%) used them more than four times. Among the 51 patients who showed improvement in dangerous behavior, 26 (51.0%) used video calls once, 8 (15.7%) used them twice, 5 (9.8%) used them three times, and 12 (23.5%) used them more than four times. The SMD for this dataset was 0.172. Of the 476 patients who showed no improvement, 227 (47.7%) used video calls once, 97 (20.4%) twice, 57 (12.0%) three times, and 95 (20.0%) more than four times. Of the 56 patients who showed improvement in instructional understanding, 22 (39.3%) used video calls once, 9 (16.1%) twice, 10 (17.9%) three times, and 15 (26.8%) more than four times. The SMD of the dataset is 0.266. The analysis suggested variability in the impact of the frequency of video calls on dangerous behaviors and instructional understanding, supporting the need for further exploration of the optimal frequency and timing of video calls to maximize patient outcomes.

**Table 4:** Video call frequency breakdown of instructional understanding and hazardous behavior SMD: Standard Mean Difference

	Risky behavior				Comprehension of instructions		
	No improvement	With improvement	SMD		No improvement	With improvement	SMD
	(n=481)	(n=51)			（n=476)	(n=56)	
video call (%)			0.172				0.266
1 time	223 (46.4)	26 (51.0)			227 (47.7)	22 (39.3)	
2 times	98 (20.4)	8 (15.7)			97 (20.4)	9 (16.1)	
3 times	62 (12.9)	5 (9.8)			57 (12.0)	10 (17.9)	
more than 4 times	98 (20.4)	12 (23.5)			95 (20.0)	15 (26.8)	

The findings from the peripheral structural model in Table 5 reveal the impact of different video call frequencies on two main factors: risk behavior and instruction comprehension.

**Table 5:** Peripheral Structure Model Odds [95% CI]: Odds ratios with 95% confidence intervals p: p-value. A p-value less than 0.05 indicates statistical significance. *: p<0.05 **: p<0.01

	Risky behavior		Comprehension of instructions	
video call	Odds [95% CI]	p	Odds [95% CI]	p
1 time	0.10 [0.07, 0.13]	<0.001	0.12 [0.09, 0.16]	**<0.001
2 times	1.10 [0.72, 1.66]	0.665	0.72 [0.47, 1.09]	0.117
3 times	1.58 [1.07, 2.34]	*0.023	0.61 [0.39, 0.94]	*0.025
more than 4 times	1.52 [1.03, 2.26]	*0.037	1.03 [0.70, 1.52]	0.866

As shown in Table 5, the frequency of video calls had a statistically significant impact on reducing risk behavior, with Exp(Coef) values of 1.58 (p=0.023, 95% CI: 1.07-2.34) and 1.52 (p=0.037, 95% CI: 1.03-2.26), respectively. As can be seen, there was an improvement in the reduction of risk behaviors in patients who made three or more video calls. However, patients who made two video calls showed no significant change in risk behaviors (Exp(Coef)=1.10, p=0.665, 95% CI: 0.72-1.66).

However, instructional comprehension improved significantly after the third video call, as suggested by the value of Exp(Coef)=0.61 (p=0.025, 95% CI: 0.39-0.94). This improvement was not observed in the second or subsequent video calls, suggesting that comprehension had no significant effect.

When the duration of hospitalization was limited to three weeks or less (see Table 6), the impact of increasing the frequency of video calls on reducing risk behaviors increased significantly. Specifically, patients who made three or more video calls showed significant improvement in reducing risk behaviors, reflected in Exp(Coef) values of 2.38 (p<0.001, 95% CI: 1.55-3.67) and 1.68 (p=0.022, 95% CI: 1.08-2.63), respectively. Conversely, the effect of the second video call was not statistically significant (Exp(Coef)=0.72, p=0.226, 95% CI: 0.43-1.22).

**Table 6:** Peripheral structure model with hospitalization limited to three weeks or less Odds [95%CI]: Odds ratios with 95% confidence intervals p: p-value. A p-value less than 0.05 indicates statistical significance. *: p<0.05 **: p<0.01

	Risky behavior		Comprehension of instructions	
video call	Odds [95% CI]	p	Odds [95% CI]	p
1 time	0.10 [0.07, 0.14]	**<0.001	0.12 [0.08, 0.16]	**<0.001
2 times	0.72 [0.43, 1.22]	0.226	0.85 [0.53, 1.37]	0.506
3 times	2.38 [1.55, 3.67]	**<0.001	1.12 [0.71, 1.76]	0.633
more than 4 times	1.68 [1.08, 2.63]	*0.022	0.97 [0.61, 1.54]	0.900

When the duration of hospitalization was limited to three weeks or less, the change in instructional comprehension due to the frequency of video calls was not significant.

## Discussion

During the COVID-19 pandemic, our study revealed that, under visitation restrictions in acute care hospitals, video calls proved to be an effective alternative to in-person visits. These video calls contributed to a reduction in the patients' risk behaviors and improved their understanding of instructions. Notably, hospital stays were within three weeks, and patients who engaged in video calls three or more times exhibited a statistically significant improvement in their risk behaviors.

Previous research has validated the efficacy of video calls in acute-care settings. Specifically, the use of recorded family video messages as a non-pharmacological nursing intervention for patients with delirium has been shown to significantly lower the incidence of delirium [22]. Furthermore, messages recorded from family members’ familiar voices have been found to be effective in reducing delirium [23]. These earlier findings reinforce the evidence suggesting that video call visitations play a crucial role in patient management, especially when there are visitation restrictions.

According to the current study, no statistically significant difference in comprehension of instructions was observed when the hospitalization period was limited to three weeks which could be due to multiple factors. A longer hospitalization period may have contributed to the stabilization of the patient's condition, adaptation to the hospital environment, and improvement in activities of daily living as a result of rehabilitation, which may have contributed to improvement in the patient’s comprehension of the instruction. However, the benefits of three or more video calls within a three-week hospital stay underscore the significance of this study. These results provide a strong rationale for introducing video calls into hospitals’ daily operations. Video calls not only support patient and family communication but also contribute to increased patient satisfaction [24]. The implementation of video calls should be actively considered as a useful tool for improving hospital visitation policies during future pandemics.

This study has several limitations. First, the study was conducted in a single acute care hospital in Fukuoka, which limits its applicability when taking the results to other healthcare settings and different cultural contexts. Further, the focus on the impact of Skype video call-based visitation did not provide insight into other communication methods and platforms. Factors such as the quality, length, and emotional content of video calls, which could significantly influence the results, have not been fully explored. The facilities studied prohibited the use of cell phones in the hospital, with the exception of some compartments, because of their potential to affect the operation of medical equipment. However, it is not known whether they personally used cell phones to conduct video calls with family members.

Second, the selection of patients for the study did not involve a randomized controlled trial, which could over-reflect certain demographic attributes and introduce selection bias. In addition, limitations of the tools used to assess the effectiveness of the intervention may have resulted in a certain bias in the results. It is also possible that confounding factors not identified during the research process may have influenced the results and their interpretation. In the future, it will be important to take these factors into account and explore optimal visitation methods using video calls, especially after a pandemic.

## Conclusions

This study determined the impact of the frequency of video calls with family members as an alternative to restricted visitation on patients' risk behaviors and understanding of instructions in a single acute care hospital during a COVID-19 pandemic. Results showed that increasing the frequency of video calls to three or more times significantly reduced patient risk behaviors when the length of hospital stay was limited to three weeks or less. These findings have important implications for clinical practice and provide a compelling case for incorporating video calls into regular visiting practices to prepare for new pandemic hospital visitation restrictions.
